# The innate immune stimulant Amplimune® is safe to administer to young feedlot cattle

**DOI:** 10.1111/avj.13156

**Published:** 2022-02-27

**Authors:** AL Alexander, E Doyle, AB Ingham, I Colditz, G McRae, S Alkemade, MP Cervantes, BC Hine

**Affiliations:** ^1^ The University of New England Armidale New South Wales 2350 Australia; ^2^ CSIRO Agriculture and Food F.D. McMaster Laboratory New England Hwy Armidale New South Wales 2350 Australia; ^3^ CSIRO Agriculture and Food Queensland Biosciences Precinct 306 Carmody Rd St Lucia Queensland 4067 Australia; ^4^ NovaVive Inc. 15 Dairy Avenue Napanee Ontario K7R 1M4 Canada

**Keywords:** Amplimune, cattle, respiratory disease, trained immunity

## Abstract

**Background:**

Infectious disease has a significant impact on livestock production. Availability of alternatives to antibiotics to prevent and treat disease is required to reduce reliance on antibiotics while not impacting animal welfare. Innate immune stimulants, such as mycobacterium cell wall fractions (MCWF), are used as alternatives to antibiotics for the treatment and prevention of infectious disease in a number of species including cattle, horses and dogs. This study aimed to evaluate the safety of Amplimune®, an MCWF‐based immune stimulant, for weaner Angus cattle.

**Methods:**

On day −1 and 0, sixty mixed‐sex Angus weaner cattle were transported for 6 h before being inducted and housed in a large single pen, simulating feedlot induction conditions. The cattle were assigned to one of six treatment groups (n = 10 per group): 2 mL Amplimune intramuscularly (2IM); 2 mL Amplimune subcutaneously (2SC); 5 mL Amplimune intramuscularly (5IM); 5 mL Amplimune subcutaneously (5SC); 5 mL saline intramuscularly (SalIM) and 5 mL saline subcutaneously (SalSC) on day 0 following transportation. Body temperature, body weight, concentrations of circulating pro‐inflammatory cytokines (TNFα, IL‐1β, IL‐6 and IL‐12) and haematology parameters were measured at various times up to 96 h post‐treatment.

**Results:**

No adverse effects from Amplimune treatment were observed. Amplimune induced an increase in circulating cytokine TNFα concentrations, total white blood cell count and lymphocyte counts indicative of activation of the innate immune system without causing an excessive inflammatory response.

**Conclusions:**

Results confirm that Amplimune can be safely administered to beef cattle at the dose rates and via the routes of administration investigated here.

**Abbreviations**
BRD: bovine respiratory disease
DNA: deoxyribonucleic acid
ELISA: enzyme‐linked immunosorbent assay
h: hours
HCT: haematocrit
IL‐12: interleukin 12
IL‐1β: interleukin 1 beta
IL‐6: interleukin 6
IM: intramuscular
LYM: lymphocyte
MCWF: mycobacterium cell wall fraction
NEU: neutrophil
NEU:LYM: neutrophil:lymphocyte ratio
SC: subcutaneous
SCFP: *Saccharomyces cerevisiae* fermentation products
TNFα: tumour necrosis factor alpha
WBC: white blood cell

Disease is a major cost to the beef cattle feedlot industry in Australia.^1^ The Australian feedlot industry is investing in research to reduce disease incidence, reduce reliance on antibiotics and improve animal health and welfare outcomes.^2^ Bovine respiratory disease (BRD) is responsible for the majority of veterinary treatments and health‐related mortalities in Australian feedlots.^3^ It is estimated to reduce profits by between $67.10 and $1647.53 per animal if there are subclinical or clinical symptoms, or the animal dies.^3^ Stress is well documented to have a suppressive effect on the immune response in livestock.^4^ Consequently, stressors imposed by transportation, induction procedures at a feedlot, change in diet and surroundings, increased human handling and co‐habitation with unfamiliar animals are all classified as risk factors that predispose cattle to BRD.^4^, ^5^, ^6^, ^7^ In addition, factors such as breed, weight and season in which cattle are introduced to the feedlot can affect their risk of BRD.^8^ Feedlot productivity and profitability could benefit from strategies to reduce the incidence and reduce reliance on antimicrobials to treat disease. Commercial vaccines are available in Australia to protect cattle against the major causal pathogens of BRD including *Mannheimia haemolytica*, bovine viral diarrhoea virus and bovine herpes virus type 1. Although these vaccines are effective at reducing the incidence of BRD associated with their target pathogens,^9^ BRD is the result of a complex opportunistic infection with mixed aetiology.^5^, ^6^ Therefore, it is difficult to provide complete protection against BRD through vaccination. Furthermore, in 2010, Australian feedlot operators reported that only 7% of cattle entering feedlots were pre‐vaccinated against *M. haemolytica* and only 0.2% were pre‐vaccinated against bovine herpes virus‐1.^9^ Though the proportion of animals pre‐vaccinated against BRD entering Australian feedlots has thought to have increased in the last decade, as suggested by a rise in seropositivity to bovine herpes virus‐1 at feedlot entry from 0.2% to 13.5%, it remains the case that a significant proportion of cattle entering Australian feedlots receive their first BRD vaccination at feedlot induction.^10^ For cattle that are not pre‐vaccinated, it is common practice for feedlot operators to vaccinate against BRD at induction. However, there are limitations to the ability of vaccination at induction to provide full protection against BRD. Firstly, there is a variable time lapse between vaccination and the establishment of protective responses, leaving a gap in which cattle are not fully protected. Secondly, targeting every pathogen associated with BRD via vaccines has not yet been achieved and may not be achievable given the complexity of the infection.

Currently, Australian feedlot operators rely on antibiotics to treat BRD.^6^, ^11^ Although evidence to suggest any significant contribution of antibiotic use in agriculture to the rise in antibiotic resistance in human pathogens is lacking, the use of antibiotics in food‐producing animals is coming under increasing scrutiny.^12^ Some consumers of animal protein are concerned by a perceived association between the use of antibiotics in livestock production and the rise in antibiotic resistance.^12^ If consumers become dissatisfied with a component of a product, demand for that product decreases, damaging those markets. Despite this, there is increasing worldwide demand for animal‐based protein, which is expected to drive a predicted 67% increase in the use of antibiotics in livestock production systems by 2030.^12^ Zelnate®, an immune stimulant based on bacterial DNA and cationic lipids, is registered for the treatment of and to reduce mortality and lung lesions associated with BRD in America,^13^ but there is no current alternative to antibiotics registered for use in Australia to prevent or treat BRD. Additional alternatives to antibiotics to prevent and treat disease in livestock will help reduce the predicted increase in antibiotic use. Thus, reducing antibiotic use is a high priority for production animal industries worldwide. To achieve this goal suitable strategies will be required to ensure animal health and welfare are not compromised due to reduced antibiotic use.

To achieve a reduction in the use of antibiotics in livestock industries whilst maintaining the highest level of welfare possible, a multi‐pronged approach is required, with a combined focus on improvements in genetics, management and the production environment. This entails breeding animals better able to resist and be resilient to disease; developing management strategies to improve the resilience of production animals; and modifying the environment to reduce pathogen exposure. Initiatives are already underway in each of these areas. Research is being done to improve the overall disease resistance of livestock by selecting animals with enhanced immune competence or general immune system performance^2^, ^14^; the efficacy of current vaccines is being improved through the use of new adjuvants like glycoprotein subunits^15^; and livestock producers are adapting the way animals are handled in order to reduce pathogen exposure.^5^ In addition, many different innate immune stimulants are being investigated for their ability to enhance immune system health, and growth and production.^16^, ^17^ The action of these immune stimulants has been linked with the concept of trained immunity. Trained immunity describes a recently discovered ability of innate immune cells to be ‘trained’ through repeated exposure to the same pathogen.^18^ The ability of innate cells to be trained was thought to be restricted to adaptive immune cells.^18^ Research has demonstrated the ability of innate immune cells to adapt to multiple exposures to various stimulants or infections via epigenetic changes, leading to a persistent, enhanced function.^18^ In this trained state, the innate immune system has a heightened ability to deal with a wide range of pathogens that the host may encounter.^19^ Armed with this new knowledge, researchers have been investigating the potential for innate immune stimulants, including *Mycobacterium bovis* and β‐glucan from *Saccharomyces cerevisiae*, to be used to provide short‐term protection against a broad range of diseases.^20^, ^21^ Indeed, trained immunity has been shown to be effective in stimulating the immune system to help prevent and treat a variety of diseases in farm animals.^21^, ^22^, ^23^

Amplimune® is a commercially available innate immune stimulant, based on mycobacterium cell wall fractions (MCWF) of *Mycobacterium phlei*, a non‐pathogenic mycobacterium found in abundance in the natural environment.^24^ The product is manufactured and marketed by NovaVive Inc. in the U.S., Canada and New Zealand^25^ but is not commercially available in Australia. Amplimune has the potential to prevent and treat a variety of diseases in food‐producing animals, particularly during periods of stress when immune system function can be compromised. Specifically, Amplimune has the potential to be used in feedlot cattle to enhance protective responses to BRD pathogens and other diseases in the high‐risk period between induction and when protection from vaccination is achieved. Despite prior use in cattle, studies on safety, dose and route of administration have not been reported in cattle of a weight and age class typical of cattle entering Australian feedlots. Therefore, this study was designed to determine safe dose rates and routes of administration for Amplimune in weaned beef cattle. We hypothesised that administration of Amplimune would induce an innate immune response in weaners but would not induce any significant adverse side effects such as severe fever, excessive systemic inflammatory responses, or significant body weight loss.

## Materials and methods

### Overview

**Figure 1:**
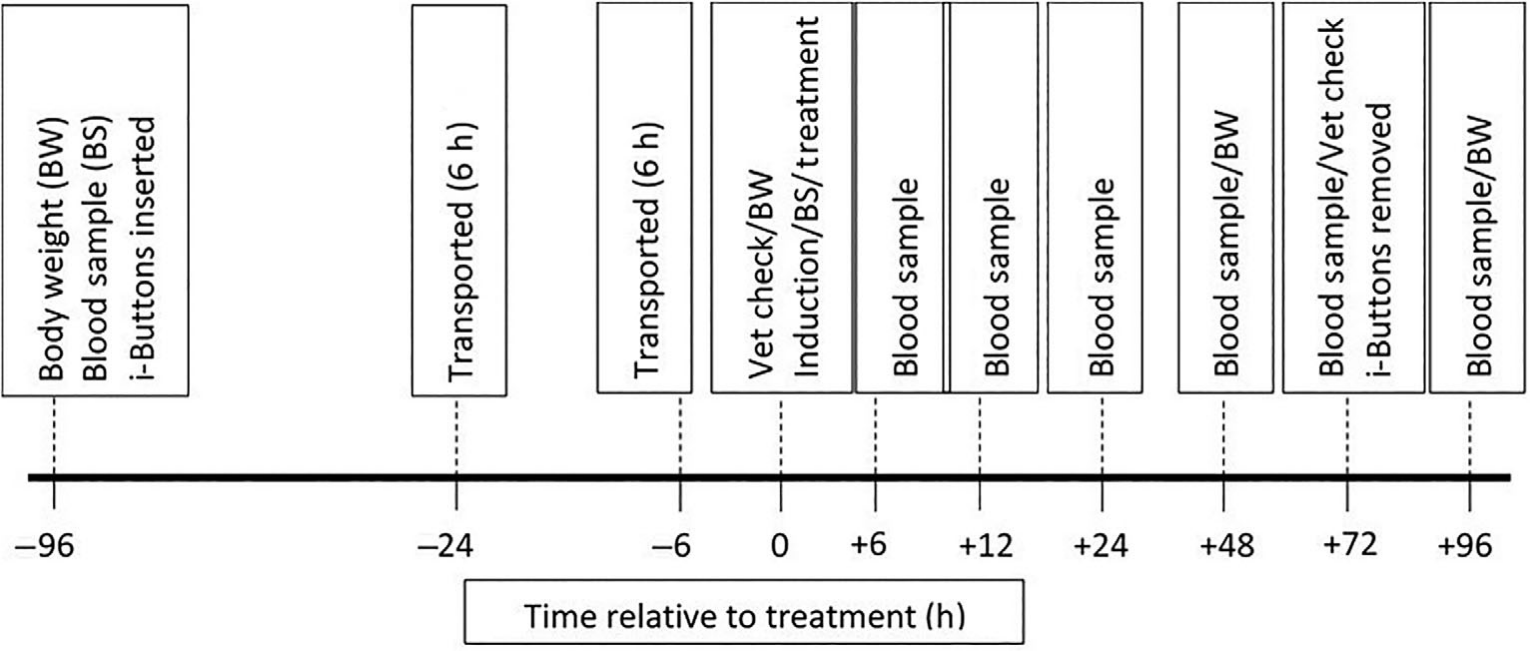
Timeline of procedures conducted including body weight (BW), blood sampling (BS), insertion and removal of i‐Buttons, transportation, veterinarian (Vet) checks, induction procedures and treatment.

This study was conducted at the CSIRO, F.D McMaster research station located near Armidale in New South Wales, Australia. All experimental procedures were pre‐approved by the CSIRO, Chiswick Animal Ethics Committee (Animal Research Authority 18/26). Experimental conditions were designed to mimic those experienced by cattle during induction into a commercial feedlot, including exposure to transportation, common animal health treatments, introduction to a commercial starter ration and confinement to a pen. Changes in body weight, core body temperature, haematology and serum pro‐inflammatory cytokine parameters induced by administration of the innate immune stimulant Amplimune, via different routes (intramuscular or subcutaneous) were measured to assess safe doses and routes of administration of the product. A detailed timeline of experimental procedures is outlined (Figure 1).

### Animals and experimental design

Sixty mixed sex Angus weaner cattle, (36 heifers and 24 steers) 6–8 months of age, were enrolled in the study. Cattle had an average body weight of 217 kg (SD = 29 kg). Cattle were grouped based on sex and body weight and then randomly allocated to six different treatment groups (n = 10). Treatments were applied high on the left side of the neck; 2 mL Amplimune (NovaVive Inc., Napanee, Ontario, Canada) via intramuscular (IM) injection (2IM), 2 mL Amplimune via subcutaneous (SC) injection (2SC), 5 mL Amplimune via IM injection (5IM), 5 mL Amplimune via SC injection (5SC), 5 mL of saline (Baxter Healthcare Pty Ltd, Old Toongabbie, NSW, Australia) via IM injection (SalIM) or 5 mL saline via SC injection (SalSC). Groups receiving saline acted as controls. To ensure treatments were not biased by sampling time, cattle were assigned a number (1–60) which was painted on their side for ease of identification and used to determine the order in which cattle were processed for sample collection. Two cattle from each of the six treatment groups were randomly assigned numbers 1–12, and so on up to 60 and were processed in numerical order for all experimental procedures.

### Transport, induction and housing

All cattle were transported by road for 6 h, (−24 to −18 h relative to treatment) and then yarded overnight with access to lucerne hay and water. The following day animals were again transported by road for 6 h (−6 to 0 h relative to treatment). Following transportation, all cattle were assessed for body condition, respiration rate, rectal temperature and heart rate by a veterinarian to ensure they were in good health. Cattle received a series of health treatments commonly administered at an induction in a commercial feedlot. These included a clostridial vaccination (UltraVac® 7in1, Zoetis) and anthelmintic treatment (Genesis Ultra, Ancare). All health treatments were administered as per manufacturers' recommendations. Treatments (Amplimune or saline) were administered as part of the induction procedures (0 h) and cattle were weighed. Cattle were closely monitored by a veterinarian for 2 h following treatment for signs of acute adverse reactions to the treatment. For the duration of the trial cattle were housed in a single large pen at a stocking density of 5.3 m^2^ per head. Cattle had access to water *ad libitum* and were fed once daily with a commercial feedlot starter ration (Tullimba Feedlot, Torryburn, NSW, Australia) at an initial rate of 3 kg/head/day, increasing incrementally to 6 kg/head/day by the fourth day. Cattle were assessed for body condition, respiration rate, rectal temperature, evidence of injection site lesions and heart rate at 72 and 96 h post‐treatment to ensure they remained in good health.

### Body temperature and body weight change

To assess changes in core body temperature induced by treatment, cattle were fitted with rectal probes, as described previously,^26^ which housed i‐Button temperature loggers (Thermocron, Castle Hill, NSW, Australia) 4 days prior to treatment (−96 h). Loggers recorded core body temperature every 5 min from −96 h pre‐treatment to 72 h post‐treatment. Temperatures recorded whilst cattle grazed undisturbed in a small paddock prior to treatment (−72 to −48 h relative to treatment) were used to establish a baseline diurnal temperature profile for individual animals. Body weight was measured at −96 h pre‐treatment to provide a baseline, on day of treatment (0 h), 48 and 96 h post‐treatment. Body weights were recorded using a calibrated weighing platform (Tru‐Test, Datamars Ltd, Banyo, QLD, Australia).

### Sample collection

Serial blood samples were collected via jugular venepuncture for haematology and to determine serum cytokine concentrations at 0, 6, 12, 24, 48, 72 and 96 h relative to treatment. Blood for haematology was collected into ethylenediaminetetraacetic acid (EDTA) vacutainers (Becton Dickinson, UK), mixed, and immediately placed on ice. In the laboratory, samples were equilibrated to room temperature, mixed well and immediately analysed on an automated haematology analyser (Cell‐Dyn 3500R, Abbott Diagnostics, Macquarie Park, NSW, Australia), using a veterinary package specifically designed for analysis of bovine samples. Parameters measured included total white blood cell counts (WBC), differential neutrophil (NEU) and lymphocyte (LYM) counts, haematocrit (HCT), and neutrophil:lymphocyte ratio (NEU:LYM) was calculated. Serum was prepared from blood collected in serum vacutainers by centrifugation at 700 *g*, for 20 min, at room temperature, and stored in multiple aliquots at −80°C for subsequent cytokine assays.

### Cytokine assays

Enzyme‐linked immunosorbent assays (ELISAs) were conducted to determine changes in the serum concentration of the pro‐inflammatory cytokines tumour necrosis factor alpha (TNFα), interleukin 1 beta (IL‐1β), interleukin 6 (IL‐6) and interleukin 12 (IL‐12). Bovine‐specific commercial ELISA kits (Bovine TNFα DuoSet ELISA, R&D Systems, Minneapolis, MN; Bovine IL‐ 6 DuoSet ELISA, R&D Systems, Minneapolis, MN) or in‐house developed assays (IL‐1β, IL‐12) were used as described previously.^27^ Circulating IL‐12 cytokine concentrations were only assessed in a subset of samples collected from cattle in the 2SC, 5SC and SalSC treatment groups at −96, 0, 6, 12, 24 and 48 h. For each animal, samples collected pre‐ and post‐treatment were run on the sample plate to minimise the effects of plate‐to‐plate variation. All assays were performed in triplicate with pooled bovine serum samples used as controls to standardise results across plates. Cytokine concentrations in samples were calculated using a standard curve generated using a series of known cytokine standards.

### Statistical analysis

Statistical analysis was conducted using Rx64 3.5.0.^28^ For parameters measured over time, repeated measures linear mixed models, fitting animal as a random effect, were used to estimate variance components using the REML method. For parameters measured only once, simple linear models were used to estimate variance components using the REML method. Residuals generated from models were tested for normality by assessing skewness and kurtosis and data transformed were required to improve normality. Data for WBC, LYM and NEU were log transformed. Data for TNFα were square‐root transformed. Tukey post hoc tests were used to evaluate multiple pairwise comparisons of group means where appropriate.

#### Haematology, cytokine and body weight

Fixed effects assessed in all models included treatment, body weight, sex and treatment*sex. For analysis of repeated measures, time and time*treatment were also fitted as fixed effects. Where appropriate, baseline parameters for body weight, cytokine concentration and haematology values measured at −96 h were fitted as covariates in statistical models. Fixed effects were removed from the final model when they were clearly not significant (P > 0.1 for single effects or P > 0.2 for interactions). Where significant treatment effects were observed, specific linear contrasts were undertaken to compare Amplimune versus saline (placebo) delivered via the same route of administration (ie. SC or IM). Specific treatment group contrasts investigated were: 2SC versus SalSC, 5SC versus SalSC, 2SC versus 5SC, 2IM versus SalIM, 5IM versus SalIM, 2IM versus 5IM. For the tabular representation of results, least squares means (LSMs) were generated from models using raw untransformed data. However, the significance of fixed effects was determined using transformed data.

#### Core body temperature

To investigate treatment effects on body temperature, the mean and maximum values logged over 6, 12, 24 and 48 h post‐treatment were calculated for individual animals. Fixed effects included treatment, body weight, sex and treatment × sex. Mean and maximum temperature data recorded during the corresponding 6, 12, or 24 h periods prior to treatment (baseline diurnal body temperature) were also calculated and values fitted as covariates in statistical models. Data from the 24 h baseline measurement period was also fitted as a covariate when analysing body temperature data recorded in the 48 h period post‐treatment. Therefore, body temperature parameters analysed were mean body temperature – 6 h (mean 6 h), 12 h (mean 12 h), 24 h (mean 24 h) and 48 h (mean 48 h) post‐treatment and maximum body temperature – 6 h (max 6 h), 12 h (max 12 h), 24 h (max 24 h), 48 h (max 48 h) post treatment.

## Results

The significance of covariate, fixed effects and interactions for all parameters in each model are summarised in Table 1.

**Table 1:** Significance (p‐values) of covariate, fixed effects and interactions for all parameters

Parameter	Trans	Cov	Trt	Weight^b^	Sex	Time	Trt × time	Trt × sex	Trt × sex × time
CBT									
Mean 6 h	None	<0.001	0.296	0.563	0.340	NA	NA	0.187	NA
Max 6 h	None	<0.001	0.498	0.312	0.043	NA	NA	0.059	NA
Mean 12 h	None	<0.001	0.679	0.143	0.703	NA	NA	0.073	NA
Max 12 h	None	<0.001	0.527	0.213	0.199	NA	NA	0.235	NA
Mean 24 h	None	0.002	0.384	0.181	0.234	NA	NA	0.794	NA
Max 24 h	None	<0.001	0.118	0.041^c^	0.247	NA	NA	0.026^c^	NA
Mean 48 h	None	<0.001	0.155	0.165	0.079	NA	NA	0.228	NA
Max 48 h	None	<0.001	0.031^c^	0.016^c^	0.056	NA	NA	0.004^c^	NA
Cytokines									
TNFα	Sq Rt	<0.001	0.605	0.891	0.617	0.692	0.003^c^	0.598	0.164
IL‐1β	Log	<0.001	0.706	0.819	0.956	<0.001	0.934	0.343	0.727
IL‐6	Log	<0.001	0.504	0.514	0.975	0.346	0.115	0.324	0.108
IL‐12^a^	Log	<0.001	0.531	0.902	0.536	0.317	0.579	0.503	0.848
Haematology									
WBCs	Log	<0.001	0.429	0.302	0.307	<0.001	0.776	0.024^c^	0.525
NEU	Log	0.072	0.100	0.753	0.184	<0.001	0.608	0.007^c^	0.873
LYM	Log	<0.001	0.131	0.618	0.527	<0.001	0.761	0.009^c^	0.234
NEU:LYM	None	<0.001	0.354	0.302	0.576	<0.001	0.759	0.010^c^	0.549
HCT	None	<0.001	0.521	0.427	0.207	<0.001	0.934	0.686	0.328
BW									
Weight	None	<0.001	0.177	NA	0.004	<0.001	0.138	0.183	0.848

^a^ Serum cytokine IL‐12 assays were only conducted on blood samples collected from 0 to 48 h post‐treatment.

^b^ Body weight recorded at −96 h (relative to treatment) was used as a fixed effect for body temperature, serum cytokine concentration, haematology and body weight parameters.

^c^ Fixed effects and interactions of interest.

Body temperatures recorded between −72 to −48 h (relative to treatment) were used as covariate for the body temperature parameter. Serum cytokine concentrations and haematology parameters recorded at −96 h (relative to treatment) were used as covariates for serum cytokine concentration and haematology parameters.

BW, body weight; CBT, core body temperature; Cov, covariate; HCT, haematocrit; IL‐1β, interleukin one beta; IL‐6, interleukin six; IL‐12, interleukin twelve; LYM, lymphocytes; Max, maximum; NEU, neutrophils; NEU:LYM, neutrophil:lymphocyte ratio; Sq Rt, square root; TNFα, tumour necrosis factor alpha; Trans, transformation; Trt, treatment; WBC, white blood cells.

### Clinical observations

No changes in clinical parameters (body condition, respiration rate, rectal temperature or heart rate) were observed in any cattle following treatments administered in the trial. Further, there was no evidence of lesions at injection sites at 2, 72 and 96 h following treatment, irrespective of treatments administered.

### Body weight

No significant differences in body weight change were detected between treatment groups over the course of the experimental period (P = 0.138).

### Serum cytokine concentrations

There was a significant treatment effect on serum concentrations of TNFα, but this effect varied over time (time*treatment interaction, P = 0.003). Results from the analysis of specific linear contrasts between Amplimune treatments and their respective controls over time are presented in Table 2. Serum cytokine TNFα concentrations were higher in 2SC treatment group animals when compared to animals in the 5SC treatment group at 24, 72 and 96 h (P = 0.04, P = 0.01 and P = 0.01 respectively) and in the SalSC control group at 72 and 96 h (P = 0.02, P = 0.04 respectively). There was a trend suggesting serum TNFα concentrations were high in 2SC treatment group animals when compared to animals in the 5SC treatment group at 48 h (P = 0.07). No significant differences were observed in serum TNFα concentrations between any treatment groups receiving treatment via IM injection. No significant differences in serum cytokine concentrations of IL‐6 or IL‐12 were observed between treatment groups. There was an effect of time on concentrations of IL‐1β (P < 0.001), however this did not vary between treatments.

**Table 2:** Treatment group LSMs (±SEM) for raw serum TNFα concentrations (ng/mL) post‐treatment

TNFα	2SC	5SC	SalSC	2IM	5IM	SalIM
0 h	3.77 ± 0.57	3.63 ± 0.57	3.65 ± 0.57	4.48 ± 0.57	3.52 ± 0.57	3.53 ± 0.58
6 h	3.02 ± 0.57	3.86 ± 0.57	4.22 ± 0.57	3.79 ± 0.57	4.05 ± 0.57	3.85 ± 0.58
12 h	3.94 ± 0.57	3.86 ± 0.57	3.48 ± 0.57	3.69 ± 0.57	4.12 ± 0.57	4.04 ± 0.58
24 h	4.92 ± 0.57^a^	3.85 ± 0.57^b^	4.13 ± 0.57^ab^	3.21 ± 0.57	3.82 ± 0.57	3.54 ± 0.58
48 h	5.16 ± 0.57	3.69 ± 0.57	3.57 ± 0.57	3.68 ± 0.57	4.02 ± 0.57	3.25 ± 0.58
72 h	5.20 ± 0.57^a^	2.52 ± 0.57^b^	2.86 ± 0.57^b^	4.27 ± 0.57	3.63 ± 0.57	3.31 ± 0.58
96 h	5.46 ± 0.57^a^	3.23 ± 0.57^b^	3.38 ± 0.57^b^	4.57 ± 0.57	3.12 ± 0.57	4.12 ± 0.58

Values in a row (within route of administration) with different superscripts are significantly different (P < 0.05). When calculating LSMs, baseline serum TNFα concentrations recorded at −96 h were fitted as a covariate in statistical models. Only treatments administered via the same route (subcutaneously (SC) or intramuscularly (IM)) were compared.

2IM, 2 mL IM injection of Amplimune; 2SC, 2 mL SC injection of Amplimune; 5IM, 5 mL IM injection of Amplimune; 5SC, 5 mL SC injection of Amplimune; h, hours; SalIM, IM injection of saline (placebo); SalSC, SC injection of saline (placebo); TNFα, tumour necrosis factor alpha.

### Haematology

Total WBC count, LYM count, NEU count, HCT and the NEU:LYM ratio changed significantly over time, independent of treatment (P < 0.001). Differences in total WBC, LYM count, NEU count and the NEU:LYM ratio were observed between treatment groups, but the effect varied depending on sex (treatment × sex interaction, P = 0.02, P = 0.01, P = 0.01 and P = 0.01, respectively). Results from the analysis of specific linear contrasts between Amplimune and control treatments within sex are presented in Table 3.

**Table 3:** Treatment group LSMs (±SEM) for raw haematology parameters, including: white blood cell, neutrophil, lymphocyte counts (×10^9^/L), neutrophil:lymphocyte ratio and haematocrit (%)

Haem	2SC	5SC	SalSC	2IM	5IM	SalIM
Heifer						
WBC	11.2 ± 0.57^a^	10.0 ± 0.58^ab^	9.17 ± 0.62^b^	10.1 ± 0.57	10.4 ± 0.57	9.61 ± 0.57
LYM	4.75 ± 0.29^ab^	5.18 ± 0.29^a^	4.14 ± 0.31^b^	4.42 ± 0.29^a^	4.59 ± 0.29^a^	3.66 ± 0.29^b^
NEU	5.29 ± 0.51	4.13 ± 0.55	4.27 ± 0.52	4.48 ± 0.51	4.50 ± 0.51	4.99 ± 0.51
NEU:LYM	1.24 ± 0.19	0.82 ± 0.19	1.35 ± 0.18	1.12 ± 0.19	1.23 ± 0.19	1.48 ± 0.18
HCT	33.0 ± 0.40	33.5 ± 0.44	33.6 ± 0.40	32.5 ± 0.40	33.3 ± 0.44	32.8 ± 0.40
Steer						
WBC	9.35 ± 0.70	10.5 ± 0.71	10.3 ± 0.70	10.2 ± 0.70^a^	8.15 ± 0.71^b^	9.46 ± 0.72^ab^
LYM	3.98 ± 0.36	4.15 ± 0.36	3.94 ± 0.36	5.15 ± 0.36^a^	4.05 ± 0.38^b^	4.63 ± 0.37^ab^
NEU	4.45 ± 0.62	5.23 ± 0.62	5.47 ± 0.62	4.00 ± 0.62	2.77 ± 0.63	3.69 ± 0.62
NEU:LYM	1.29 ± 0.23	1.60 ± 0.23	1.48 ± 0.23	0.85 ± 0.23	0.80 ± 0.23	0.83 ± 0.23
HCT	32.5 ± 0.45	33.1 ± 0.42	33.2 ± 0.44	32.1 ± 0.43	32.9 ± 0.42	32.4 ± 0.45

Values in a row (within routes of administration) with different superscripts are significantly different (P < 0.05). When calculating LSMs, baseline haematology parameters recorded at −96 h were fitted as a covariate in statistical models.

2IM, 2 mL IM injection of Amplimune; 2SC, 2 mL SC injection of Amplimune; 5IM, 5 mL IM injection of Amplimune; 5SC, 5 mL SC injection of Amplimune; Haem, Haematology parameters; HCT, haematocrit; LYM, lymphocyte count; NEU, neutrophil count; NEU:LYM, neutrophil:lymphocyte ratio; SalIM, IM injection of saline (placebo); SalSC, SC injection of saline (placebo); WBC, white blood cell count.

Following treatment, WBC counts were higher in 2SC heifers compared to control (SalSC) heifers (P = 0.02). Differences were also observed in WBC counts in steers from the 2IM and the 5IM groups with steers in 5IM having higher WBC counts post‐treatment (P = 0.04).

In contrast, following treatment LYM counts were higher in 2IM and 5IM heifers compared to control (SalIM) heifers (P = 0.03 and P = 0.02 respectively). Differences were also observed in LYM counts in 5SC heifers compared with control (SalSC) heifers, with 5SC heifers having higher LYM counts post‐treatment (P = 0.04). Differences were also observed in LYM counts in the 2IM and 5IM steers with the 2IM steers having higher LYM counts post‐treatment (P = 0.04).

As differences in LYM counts were observed in heifers receiving 5 mL Amplimune versus saline (control) when administered either IM or SC, specific contrasts were conducted to compare differences in LYM counts in heifers receiving Amplimune IM or SC at the 5 mL dose. No significant differences were observed (P = 0.25).

### Temperature

Treatment group differences were observed in maximum core body temperature at 24 and 48 h post‐treatment, however, the effect of treatment varied depending on sex (treatment × sex interaction, P = 0.03 and P = 0.004 respectively). Results from the analysis of specific linear contrasts between Amplimune treatment groups and their respective control on mean and maximum core body temperature within sex are presented in Tables 4 and S1. Steers in 2SC had higher maximum core body temperatures at 24 and 48 h post‐treatment, compared to steers in the control group (SalSC) (P = 0.05, P = 0.02 respectively). No significant differences were observed in mean core body temperature between any treatments (Table S1). There was a significant effect of sex on maximum body temperature 6 h post‐treatment (P = 0.04) and there was a significant effect of body weight on maximum core body temperature at 24 and 48 h post‐treatment (P = 0.04 and P = 0.02 respectively).

**Table 4:** Treatment group LSMs (±SEM) for maximum core body temperature (°C) within 6, 12, 24 or 48 h blocks post‐treatment

Max body temp	2SC	5SC	SalSC	2IM	5IM	SalIM
Steer						
6 h block	39.9 ± 0.18	39.6 ± 0.16	39.5 ± 0.16	39.4 ± 0.16	39.1 ± 0.17	39.5 ± 0.16
12 h block	40.3 ± 0.25	39.8 ± 0.22	39.7 ± 0.21	39.5 ± 0.21	39.2 ± 0.22	39.7 ± 0.21
24 h block	40.9 ± 0.26^a^	40.1 ± 0.22^ab^	39.9 ± 0.22^b^	39.9 ± 0.22	39.5 ± 0.22	39.8 ± 0.22
48 h block	41.2 ± 0.24^a^	40.6 ± 0.21^ab^	40.1 ± 0.21^b^	40.2 ± 0.21	39.7 ± 0.21	39.8 ± 0.21
Heifer						
6 h block	39.5 ± 0.14	39.7 ± 0.18	39.5 ± 0.13	39.9 ± 0.15	39.6 ± 0.13	39.7 ± 0.14
12 h block	39.8 ± 0.19	39.9 ± 0.25	39.8 ± 0.18	40.0 ± 0.20	39.8 ± 0.17	39.9 ± 0.19
24 h block	39.8 ± 0.20	39.9 ± 0.25	39.8 ± 0.18	40.0 ± 0.20	40.0 ± 0.18	39.8 ± 0.20
48 h block	39.8 ± 0.19	40.1 ± 0.24	40.0 ± 0.17	40.1 ± 0.19	40.2 ± 0.17	39.9 ± 0.19

Values in a row (within the route of administration) with different superscripts are significantly different (P < 0.05). When calculating LSMs, baseline body temperatures for individual cattle in 6, 12 or 24 h blocks recorded between −72 and −48 h were fitted as a covariate in statistical models. Baseline body temperatures (°C) for individual cattle in the 24 h block recorded between −72 and −48 h were fitted as a covariate in the 48 h block statistical model. Only treatments administered via the same route (subcutaneously (SC) or intramuscularly (IM)) were compared.

2IM, 2 mL IM injection of Amplimune; 2SC, 2 mL SC injection of Amplimune; 5IM, 5 mL IM injection of Amplimune; 5SC, 5 mL SC injection of Amplimune; Max, maximum; SalIM, IM injection of saline (placebo); SalSC, SC injection of saline (placebo); temp, temperature.

## Discussion

The current study investigated the response of weaner Angus cattle to the administration of Amplimune, an innate immune stimulant based on MCWF, under conditions that mimicked feedlot induction. Changes in body temperature, body weight, circulating pro‐inflammatory cytokines and haematology parameters induced by treatment with Amplimune were investigated at varying dose rates and routes of administration. Results supported the hypothesis that administration of Amplimune to weaner beef cattle would induce an immune response that is safe and does not have negative impacts on health and performance outcomes in the 96 h following administration.

Clinical examinations of all animals were done pre‐treatment, to ensure animals were healthy when treated, and again post‐treatment to identify any potential adverse effects of treatments. Normal body condition, respiration rates, rectal temperatures and heart rates were observed pre‐treatment, post‐treatment (2 h) and again at the conclusion of the trial (96 h). Results showed that several of the response parameters measured in the study were unaffected by treatment throughout the trial. These included weight gain, circulating concentrations of the pro‐inflammatory cytokines IL‐1β, IL‐6, IL‐12 and the haematology parameters neutrophil count, haematocrit and neutrophil:lymphocyte ratio. In contrast, increases in circulating concentrations of the pro‐inflammatory cytokine TNFα, WBC and LYM counts and maximum body temperatures, were induced by treatment with Amplimune. Although treatment with Amplimune resulted in significant increases in these parameters, the maximum observed values only exceeded normal reference ranges up to 72 h post‐treatment and returned to normal values by 96 h. A normal temperature range of 37.8–39.2°C has been reported in adult cattle previously although young cattle and individuals can have higher temperature ranges.^29^ Throughout the current trial, the maximum core body temperature in cattle treated with Amplimune ranged between 39.6–41.6°C, which dropped to 36–40.3°C on the final day of the trial. Similarly, cattle treated with saline (control) recorded a maximum range of 39.5–41.3°C, but by the conclusion of the trial that range had dropped to 38.8–40.6°C suggesting that treatment was not the cause of the change in temperature. Likewise, during the trial, 14 animals had lymphocyte counts which exceeded the reported normal range for bovines (2.5–7.5 × 10^9^/L).^30^ However, these animals were from all six treatment groups, including controls, and eight of the 14 had baseline lymphocyte counts outside the reference ranges prior to treatment. Lymphocyte counts, for all but one animal, returned to normal by 96 h post‐treatment. A similar trend was observed for WBC counts. In 36 animals, across all six treatment groups, WBC count increased beyond the normal reference range of 4–12 × 10^9^/L,^30^ however, 24 of these animals had baseline WBC counts outside the reference range prior to treatment. WBC counts, excluding one animal, returned to within the normal reference range by 96 h post‐treatment. To the authors' knowledge, an official normal range for concentrations of serum cytokines in cattle has not been reported, but many studies have tracked changes in concentrations of TNFα in serum from pre‐ to post‐challenge^31^, ^32^, ^33^, ^34^, ^35^, ^36^, ^37^, ^38^, ^39^, ^40^ (Table S2). The serum concentrations of TNFα across studies ranged from 0 to 140 ng/mL pre‐challenge, and 0.25 to 240 ng/mL post‐challenge. In the current study, the highest concentration of TNFα in serum observed was 22 ng/mL at 48 h post‐treatment. This indicates TNFα is within the range of previously reported baseline values and suggests that administration of Amplimune did not induce an excessive inflammatory response. Therefore, the current study suggests that MCWF is safe to administer to young beef cattle, while results from previous studies suggest it may play a role in enhancing immune system function and the ability of cattle to resist subsequent pathogen exposure.^17^, ^25^, ^41^, ^42^

Many of the same measures used to assess safety are also measures of innate immune system activation. The objective when using immune stimulants to help prevent disease is to stimulate the immune system to a level, which enhances responses to subsequent disease challenges without causing adverse effects, such as excessive inflammation. The challenge, therefore, is to choose a dose and route of administration that can enhance future immune responses without causing excessive inflammation. Examples of immune stimulants that have proven to induce excessive stimulation include recombinant bovine IL‐1β which reduced *Staphylococcus uberis* infection but induced sterile mastitis^43^ and an intramammary loop for prevention of mastitis in dairy cows which significantly increased leukocyte concentrations.^44^ Administration of Freund's complete adjuvant sometimes leads to severe lesions at the site of injection and in severe cases, mortality,^45^, ^46^ resulting in its withdrawal from widespread use as an adjuvant in laboratory animals. In the current study, MCWF in Amplimune did not cause lesions at the site of injection, nor did it induce excessive immune activation. However, there were indications of moderate immune activation including increases of the circulating pro‐inflammatory cytokine TNFα, when administered subcutaneously (SC) at a dose rate of 2 mL. Amplimune also induced increases in total WBC count in heifers when administered via SC or IM injection at a dose rate of 2 mL; and increases in lymphocyte counts in heifers when administered via SC or IM injection at a dose rate of 5 mL. In other studies investigating MCWF, from non‐Amplimune sources, in mice,^47^ dogs^48^ and horses,^49^ changes have been observed in a variety of parameters. Activation of cytotoxic T‐cells by MCWF induced the reduction of three different cancer cell lines in mice tumours^47^; clinical symptoms of bladder cancer, including dysuria, pollakiuria and haematuria, were reduced following administration of MCWF in dogs^48^; and a reduction in the number of mares positive for endometritis, based on exfoliative cytology and bacteriology, when administered MCWF at 48 h post‐experimental challenge.^49^ In these studies, immune activation was induced without excessive inflammation. Other studies have also investigated the ability of different types of innate immune stimulants to enhance immune system function in cattle. Liposome‐toll‐like receptor complexes delivered intranasally to beef calves induced significant increases in the percentage of CD14+ monocytes in nasal mucosal samples and upregulated expression of interferon‐gamma, interleukin‐8 and monocyte chemoattractant protein‐1 gene transcripts in nasopharyngeal samples.^16^ Fermentation products from the yeast *Saccharomyces cerevisiae* (SCFP) have been fed to dairy calves, reducing the incidence of diarrhoea.^23^ The effects of supplementation with SCFP in beef calves deliberately challenged with lipopolysaccharide from *Escherichia coli* have been reported with increased platelet concentrations and reduced serum TNFα concentrations observed in supplemented calves as compared with calves not fed SCFP.^33^

The specific active ingredient of Amplimune is fragmented sections of *M. phlei* cell wall with nucleic acids conserved onto it. These fragments are adjuvanted in 2% squalane, a fully hydrogenated, digestible oil that is a precursor of cholesterols. The final product is an emulsion of MCWF in squalane in phosphate‐buffered saline. The lack of pathogenicity of *M. phlei*, particularly compared to other mycobacteria, such as *M. bovis*,^50^ as well as its lack of cross‐reactivity with bovine tuberculosis and Johne's disease tests,^51^ makes it ideal for inclusion in an immune stimulant formulation for cattle.

The efficacy of Amplimune to prevent and treat disease has been demonstrated in several breeds and age classes of cattle, through the assessment of pro‐inflammatory responses as an indication of innate immune induction, and also by assessing clinical symptoms of the disease. For example, Amplimune has been used to treat the symptoms of *Escherichia coli* infection in neonatal dairy calves^41^ and has been shown to reduce morbidity and mortality, and increase weight gain in feedlot steers with an average weight of 115 kg, however, the influence of pen effects on observed results could not be assessed in the study.^25^ Amplimune has also been successfully used to prevent mastitis and arthritis in adult dairy cows.^52^ Although no significant difference in the likelihood of health treatments for both respiratory disease and/or diarrhoea was observed between calves in treatment groups receiving Amplimune or saline (control) during the first 9 weeks of life, more calves in the control group received disease treatments in the first 30 days following transportation.^17^ Administration of Amplimune to neonatal dairy calves has also been shown to enhance immune system activation.^42^ Subcutaneous injection of Amplimune in neonatal dairy calves increased the number of major histocompatibility complex Class II^+^ CD4^+^ T‐cells/mL (in peripheral blood); indicating an increase in the frequency of activated circulating lymphocytes.^42^ Conversely, subcutaneous injection of Amplimune in dairy cows reduced clinical signs of metritis and mastitis but had no effect on white blood cells.^53^ Other studies with different MCWFs have found increased production of a range of pro‐inflammatory cytokines including interleukin‐12 (IL‐12), interleukin‐6 (IL‐6), tumour necrosis factor‐alpha (TNFα) and interferon‐gamma (IFNγ), as well as increased neutrophils and primed macrophages in response to treatment.^54^, ^55^ The efficacy of MCWF from *M. phlei* to prevent and treat disease has also been investigated in horses and dogs.^48^, ^49^

Importantly, studies have also shown that treatment of cattle with *M. phlei* does not cause false‐positive results in bovine tuberculosis and Johne's disease tests, making it safe for use in food‐producing animals, without compromising disease surveillance.^51^ Results of the current study suggest that Amplimune is safe to use in young beef cattle. Further studies will be required to evaluate the potential for Amplimune to be used as an alternative to antibiotics to prevent and treat disease in beef cattle via enhancement of immune system function and activation of trained immunity. A target application is in Australian feedlot cattle to reduce the incidence of BRD, where currently the only treatment option is the use of antibiotics and ancillary anti‐inflammatory therapy.

## Conclusion and recommendations

The current study demonstrates that the innate immune stimulant Amplimune is safe to use in young feedlot cattle at 2 and 5 mL dose rates and SC and IM delivery routes. This was shown by a lack of any adverse side effects and no effect of treatment on weight gain, circulating concentrations of pro‐inflammatory cytokines IL‐1β, IL‐6 and IL‐12, and haematology parameters neutrophil count, haematocrit and neutrophil:lymphycyte ratio. Moderate innate immune induction triggered by treatment with Amplimune was demonstrated by increased total WBC count, lymphocyte count and circulating concentrations of the pro‐inflammatory cytokine TNFα. Future studies will assess the potential use of Amplimune as an antibiotic alternative and for the prevention of disease through stimulation of the innate immune response and trained immunity.

## Conflict of interest and sources of funding

The work was co‐funded by Meat and Livestock Australia (MLA) and the Commonwealth Scientific and Industrial Research Organisation (CSIRO). Author Annika Alexander was the recipient of the Ian McMaster Bequest scholarship and the QTAC Rural & Regional Enterprise scholarship. Authors McRae G, Alkemade S and Cervantes M were employees or are still employed by NovaVive (a company, which previously had or currently holds licensing rights for Amplimune™) at the time of study execution and manuscript preparation.

## Supporting information


**Table S1** Treatment group LSMs (±SEM) for Mean core body temperature (°C) within 6, 12, 24 or 48 h blocks post‐treatment.
**Table S2** Experiments in cattle with recorded serum concentrations of pro‐inflammatory cytokine TNFα, pre‐ and post‐challenge.
