# Supplementary material for: The innate immune stimulant Amplimune® is safe to administer to young feedlot cattle
Source: Aust Vet J. 2022 Feb 27;100(6):261–70. doi: 10.1111/avj.13156 (PMC9306767; doi:10.1111/avj.13156)
Supplement: Supplementary file 1 — Table S1 Treatment group LSMs (±SEM) for Mean core body temperature (°C) within 6, 12, 24 or 48 h blocks post‐treatment. Table S2 Experiments in cattle with recorded serum concentrations of pro‐inflammatory cytokine TNFα, pre‐ and post‐challenge. [file AVJ-100-261-s001.docx]

**Appendix**

**Table 1.** Treatment group LSMs ($\pm$SEM) for Mean core body temperature (°C) within 6, 12, 24 or 48 h blocks post-treatment.

| Mean body temp | 2SC | 5SC | SalSC | 2IM | 5IM | SalIM |
| --- | --- | --- | --- | --- | --- | --- |
| Steer |  |  |  |  |  |  |
| 6 h block | 39.5$\pm$0.19 | 39.2$\pm$0.16 | 39.2$\pm$0.16 | 39.1$\pm$0.16 | 38.8$\pm$0.17 | 39.3$\pm$0.16 |
| 12 h block | 39.7$\pm$0.19 | 39.3$\pm$0.17 | 39.3$\pm$0.17 | 39.2$\pm$0.17 | 38.8$\pm$0.17 | 39.3$\pm$0.17 |
| 24 h block | 39.8$\pm$0.35 | 39.2$\pm$0.30 | 39.2$\pm$0.30 | 39.3$\pm$0.30 | 38.9$\pm$0.31 | 39.2$\pm$0.31 |
| 48 h block | 40.0$\pm$0.25 | 39.4$\pm$0.21 | 39.3$\pm$0.21 | 39.3$\pm$0.21 | 38.9$\pm$0.22 | 39.2$\pm$0.22 |
| Heifer |  |  |  |  |  |  |
| 6 h block | 39.2$\pm$0.15 | 39.4$\pm$0.19 | 39.1$\pm$0.13 | 39.4$\pm$0.15 | 39.3$\pm$0.13 | 39.3$\pm$0.15 |
| 12 h block | 39.2$\pm$0.15 | 39.3$\pm$0.19 | 39.1$\pm$0.14 | 39.4$\pm$0.15 | 39.4$\pm$0.14 | 39.4$\pm$0.15 |
| 24 h block | 39.1$\pm$0.27 | 39.1$\pm$0.35 | 39.1$\pm$0.25 | 39.2$\pm$0.27 | 38.7$\pm$0.25 | 39.3$\pm$0.28 |
| 48 h block | 39.2$\pm$0.19 | 39.2$\pm$0.25 | 39.0$\pm$0.17 | 39.3$\pm$0.19 | 39.1$\pm$0.18 | 39.3$\pm$0.19 |

When calculating LSMs, baseline body temperatures for individual cattle in 6, 12 or 24 h blocks recorded between -72 and -48 h were fitted as a covariate in the corresponding statistical models. Baseline body temperatures (°C) for individual cattle in the 24 h block recorded between -72 and -48 h were fitted as a covariate in the 48 h block statistical model. Only treatments administered via the same route (subcutaneously (SC) or intramuscularly (IM)) were compared.

2IM, 2mL IM injection of Amplimune; 5IM, 5mL IM injection of Amplimune; SalIM, IM injection of saline (placebo); 2SC, 2mL SC injection of Amplimune; 5SC, 5mL SC injection of Amplimune; SalSC, SC injection of saline (placebo); temp, temperature.

**Table 2.** Experiments in cattle with recorded serum concentrations of pro-inflammatory cytokine TNFα, pre- and post-challenge.

| Cattle type | Challenge | TNFα pre-challenge | TNFα post-challenge | Source |
| --- | --- | --- | --- | --- |
| Dairy | Heat stress | ~140 ng/mL | ~240 ng/mL | 30 |
| Cattle from Egypt | Hydatid cyst disease | 9.29 ng/mL | 13.8 ng/mL | 31 |
| Dairy | Mastitis | 7.06 ng/mL | 12.5 ng/mL | 32 |
| Dairy | Heat stress | ~1.02 ng/mL | ~0.87 ng/mL | 33 |
| Feedlot calves | BRD | 0.50 ng/mL | ~2.50 ng/mL | 34 |
| Beef steers | Exposure to *M. haemolytica*+ BVDV | 0.37 ng/mL | 1.98 ng/mL | 35 |
| Dairy cows | Heat stress +/- supplement | 0.22 ng/mL | 0.20 ng/mL | 36 |
| Dairy calves | Vitamin/mineral concentrate | 0.13 ng/mL | 0.15 ng/mL | 37 |
| Beef steers | Lipopolysaccharide | ~0.00 ng/mL | ~13.0 ng/mL | 38 |
| Beef calves | Lipopolysaccharide +/- supplement | ~0.00 ng/mL | ~0.25 ng/mL | 39 |

BVDV, bovine viral diarrhoea virus
